# Software reviews

**DOI:** 10.1155/S1463924600000250

**Published:** 2000


Journal of Automated Methods & Management in Chemistry, Vol. 22, No. 5 (September–October 2000) pp. 159–161

### Software reviews

### Add-on package gives Mathematica users extraordinary graphing capabilities through a simple interface

*Northampton, MA, 13 January 2000*: Microcal™ Software, today announced the release of the *Origin Link for Mathematica*, an easy-to-use interface between Origin®, a leading scientific graphing and data analysis software, and Mathematica, the world's only fully integrated technical computing system. The *Origin Link for Mathematica* greatly simplifies the plotting, customization and export of publication-quality graphs for Mathematica users, by giving the user access to a vast number of plot types as well as the ease-of-use of a point-and-click interface for editing their graphs.

*Mathematica* provides numeric and symbolic calculations, visualization, and a complete programming environment. Used worldwide by students, engineers, physicists, analysts and other technical professionals, many have discovered *Mathematica*'s unique combination of unmatched computational power.

'Many Mathematica users have found Mathematica's plotting and graph customization capabilities slightly overwhelming, and wish for an easy way to produce publication-quality graphs,' said Joe Przechocki, Product Manager, at Microcal Software. 'The *Origin Link for Mathematica* add-on package gives them a whole new kind of plotting power, letting them perform powerful calculations within Mathematica while producing and customizing graphs in Origin, in ways they couldn't imagine before.'

Origin is a scientific graphing and data analysis software widely used by scientists and engineers around the world. With this new *Origin Link for Mathematica* interface, Mathematica users can tap into the graphing power of this exciting software. The link truly combines the computational power of Mathematica with Origin's intuitive graphing capabilities. Users will be able to manipulate and visualize data and equations with just a few mouse clicks – and then export their final graph in a wide variety of file formats such as EPS, JPG, TIF and more. Origin also acts as an OLE2 graphics server for Microsoft Windows applications.

The *Origin Link for Mathematica* consists of a multi-tabbed dialogue box that allows data and expressions to be transferred between Mathematica and Origin. Any Mathematica expression can be entered and the results can be viewed in a scrollable window. Mathematica's extensive technical computing capabilities can be used to analyse the data and the results can be graphed in Origin. Double-clicking on any graph element in Origin brings up an intuitive dialogue box that can be used to achieve the desired look with ease. Using Origin to create and edit graphs will save the Mathematica user considerable time.

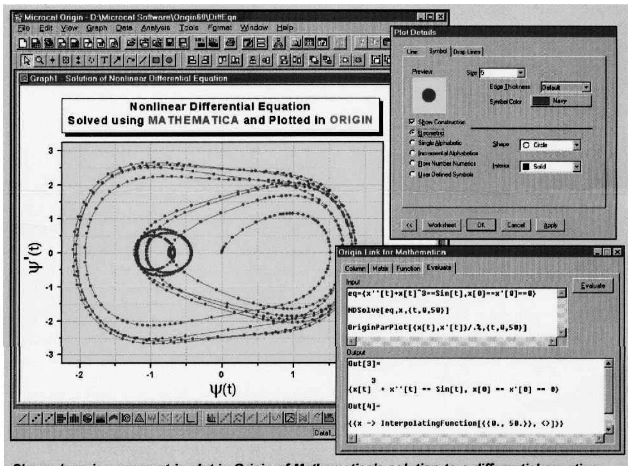

**Shown here is a parametric plot in Origin of Mathematica's solution to a differential equation. Graph elements can be easily customized using the intuitive dialog box displayed above.**

### Pricing information

$0 – free download

*Systems Requirements*

*The Origin Link for Mathematica* requires *Mathematica* 4, and *Origin* 6, both for Windows®.

*For more information on Microcal Software's other products, visit our web set at http://www.microcal.com or contact: Chay Iv, Marketing Coordinator (USA & Canada), Tel (USA): 800 969 7720; Tel (International): + 1 413 586 2013; e-mail: chay@microcal.com*

### Zymark Corporation Launches Staccato™ Workstations De-bottlenecking drug development pipeline in genomics, screening and ADME/Toxicology

*Hopkinton, MA, 16 May, 2000*: Critical bottlenecks in the drug pipeline identified in the areas of genomics, screening, compound management and ADME/toxicology are placing increased pressure on laboratories. To address these growing challenges, Zymark Corporation announces the launch of its Staccato application workstations.

These application-focused workstations, based on 20 years of automation experience coupled with innovative liquid handling, stacking and control software technologies from recently acquired Scitec, provide a uniform architecture using reliable and econmical building blocks. The Staccato workstations expand flexibility, capacity and throughput using 96-, 384- and 1536-well microplate technologies.

At the heart of the Staccato Workstations is the Zymark SciClone™ liquid handling and Presto™ autostacking technology, advanced articulated arm robotics and the new CLARA™ 2000 open architecture software. These core automation modules with optional readers, washers, incubators and filtration units give the user the capability to select the peripherals needed for the application. The result is a scalable, cost-effective workstation with a small footprint and flexibility, throughput and capacity not currently available from any other supplier.

'Two years ago, Zymark launched the Allegro Ultra High Througput Screening System which was truly a revolutionary screening breakthrough', explains Kevin Hrusovsky, Zymark's President and CEO. 'Building on this successful model for standardized automation and additional customer input, we created a broader vision to provide new laboratory automation technology to debottleneck critical applications within the drug pipeline. Since the acquisition of Scitec Automation nine months ago, Zymark has successfully incorporated new xyz and articulated arm robotics, and scheduling control software that are the cornerstone of the Staccato applications. Eight key application areas in DNA purification, amplification and clean-up as well as ADME toxicology, traditional screening, hit-picking and plate replication all use the same easy-to-use building blocks.'

'Our customers are high profile scientists in key genomic research facilities. They expressed a need for a high throughput automated workstation to purify plasmid DNA, a very common and time consuming application,' commented Roy Vallie, Vice President of Sales and Marketing at Eppendorf 5 Prime. 'Using Zymark's standard liquid handling and storage technologies along with their scheduling software, we have been able to deliver a standard DNA purification platform, compatible with our Perfect Prep-96 chemistry, that reliably delivers 10 times more throughput than any automated platform available on the market – and more importantly is out of the box and running in a fraction of the time. Response from customers currently using the workstation has been amazing.'

Zymark Corporation, headquartered in Hopkinton, MA, is a worldwide leader in laboratory automation for pharmaceutical and biotechnology applications. Zymark serves the worldwide pharmaceutical and biotechnology industries, and hosts the annual ISLAR conference on laboratory automation. The company employs about 300 people in the USA, Canada, Japan and Europe, and has authorized sales and service distributors throughout the rest of the world.

*For more information contact: Lynda Thomas, Marketing Communications, Zymark Corporation, Hopkinton, MA 01748, USA. Tel: + 1 508 497 6549; e-mail: lynda.thomas@zymark.-com; www.zymark.com.*

### Alias announces I-Sketch Field, the worlds first piping isometric software on a palm PC

Alias Limited, market leader for piping isomeric software, has announced the introduction of I-Sketch Field, the first ever piping isometric software to be made available on a Palm PC. This innovative product has been specifically designed to be used by engineers who are looking for a portable solution to carry out design alterations and capture as-built piping information on-site.

I-Sketch Field offers an easy to use, compact and productive application that allows the verification of existing pipe configurations and the creation of new isometric drawings for tie-ins. Verification can be performed by loading the existing pipes, from any 3D model supporting ISOGEN, onto the hand-held device. Comments and notes are then added for later editing or inclusion into the 3D model.

Synchronization between I-Sketch Field and I-Sketch enables all information and adaptations made on the Palm PC during the site visit to be easily and quickly transported back into I-Sketch installed on the PC. Other tools within I-Sketch can then transfer all the material back into your Plant Design System including PDS, PDMS.

I-Sketch represents breakthrough technology that is sold at a very competitive price. Isometric drawings can be created in minutes from simple sketches using the industry de-facto ISOGEN. It is so easy to use that specialist drafting, CAD or 3D modelling skills are no longer required.

I-Sketch, together with I-Sketch Field offers the latest in engineering technology, delivering easy to use, appropriate tools for plants engineers and piping designers.

### More on Alias

Alias Limited is based in the north west of England and specializes in the development of software for the process industries. The core competence of the company is automating labour-intensive operations in plant design with a particular focus on pipes.

Alias leads the world for automatic piping isometric generation with their flagship product, ISOGEN, which is available from Intergraph, Rebis, Bentley, CADCentre, Kockums Computer Systems, Aquaconsult, CEA International, Profox, COADE, Orange Systems, PRO-CAD, Unigraphics and Parametric Technology Corporation. More recent additions to the Alias product portfolio include SPOOLGEN for the production and management of fabrication spool isometrics and I-Sketch for the automatic production of piping isometrics from simple sketches.

The company was formed in 1991 by a team who had been involved in these activities within ICI plc. The company's products are now available worldwide via a comprehensive network of resellers.

### Alias Limited release I-Sketch Version 2

Alias Limited has announced the availability of I-Sketch Version 2. This new release offers all the improvements that existing users have requested, whilst maintaining the quality and ease of use of Version 1.3.

I-Sketch Version 2 incorporates many new features and enhancements that will raise productivity, provide greater usability and increase efficiency when creating and editing isometric drawings. Improvements include the Import Utility, dimensioning enhancements, greater pipe editing capabilities, extra catalogue and specification properties, and design rule checking to minimize drawing errors.

The import utility will allow any pipe created in a plant design system, that supports ISOGEN, to be imported into I-Sketch for editing purposes. Pipe editing has been made easier with the ability to edit multiple components and apply global changes. Automatic dimensioning reduces the time it takes to prepare the sketch and create the isometric drawing by evaluating known information and applying it to other sections of the piping network. Enhancements to the specification system ensure that every component will automatically have an item code assigned to it and thus be fully specified when you place it on the sketch.

I-Sketch version 2 is a complete package that simplifies the task of creating isometric drawings by non-CAD-trained engineering personnel.

Software is currently shipping to selected users for final beta testing. The full production version is expected to be available mid-March 2000.

*For more information contact: Andy Osborne/Rebecca Forrest, Alias Limited, Tel: + 44 (0) 1928 579 311; Fax + 44 (0) 1928 579 389; e-mail: andy.osborne@alias.ltd.uk; rebecca.forrest@alias.ltd.uk.*

*More information about Alias is available at http:/www.alias.ltd.uk*

